# Revealing the Sound Transmission Loss Capacities of Sandwich Metamaterials with Re-Entrant Negative Poisson’s Ratio Configuration

**DOI:** 10.3390/ma16175928

**Published:** 2023-08-30

**Authors:** Fangyi Li, Yuanwen Chen, Dachang Zhu

**Affiliations:** School of Mechanical and Electrical Engineering, Guangzhou University, Guangzhou 510006, China; 2112107044@e.gzhu.edu.cn

**Keywords:** sound transmission loss, sandwich structure, negative Poisson ratio, acoustic metamaterial

## Abstract

Due to the influence of mass law, traditional lightweight sandwich structures have struggled to surpass solid structures in sound insulation performance. To this end, we propose an acoustic metamaterial structure with a sandwich configuration based on the re-entrant negative Poisson’s ratio (NPR) structure and systematically investigate its sound transmission loss (STL) performance under incident plane wave conditions. We used the acoustic impedance tube method to experimentally study the sound insulation performance of the re-entrant NPR sandwich structure under free boundary conditions, and then established an acoustic analysis simulation model based on COMSOL Multiphysics software, which verified that the results obtained by the experiment and the numerical simulation were in good agreement. The results show that the sandwich structure exhibits excellent sound transmission loss performance in the studied frequency range (250–4000 Hz), and the overall sound insulation performance exceeds the curve of the mass theorem, basically achieving more than 20 dB when the sandwich thickness is 2 cm. Finally, we conduct parametric studies to establish a correlation between the geometric design of NPR sandwich structures and their sound transmission loss performance. The research shows that the changes of the length of the ribs, the distance from the ribs to the center of the unit, and the length of the upper wall and the lower wall have a significant impact on the sound insulation performance of the re-entrant NPR sandwich structure, while the change of the wall thickness basically will not affect the sound insulation performance of the sandwich structure. This research can provide practical ideas for the engineering application of noise suppression designs of lightweight structures.

## 1. Introduction

Noise suppression is of utmost importance in various fields, including aerospace, maritime transportation, and high-speed trains. Especially during the operation of high-speed trains, the sound wave energy is concentrated in the 250–4000 Hz noise frequency band, which will have adverse effects on human health [1,2,3].

Due to the easily parameterized and modifiable nature of the sandwich structure, the material characteristics can be flexibly changed, and the acoustic wave and elastic wave properties can be changed in space and frequency [4,5,6]. This has garnered significant attention from both the academic and industrial sectors, and it has been applied in various fields, including high-speed manned transportation [7,8,9,10,11,12,13,14]. Despite extensive exploration of sandwich structures in sound insulation, numerous achievements made, such as the metal lattice sandwich structure and the pyramid sandwich structure [15,16,17,18,19,20,21,22,23,24]. However, the sound transmission loss performance of the traditional sandwich structures currently fall slightly short in comparison to the sound insulation performance of solid core structures with equivalent volume. It is difficult for the sound insulation to match or surpass the mass law curve. Therefore, there is a strong desire for breakthroughs in this area [25,26]. In pursuit of this goal, efforts are being directed towards the development of novel lightweight sandwich acoustic metamaterials, aiming to enhance the sound transmission loss performance of sandwich structures and striving for an early breakthrough beyond the mass law.

The negative Poisson’s ratio sandwich structure has emerged as a promising metamaterial for sound insulation due to its unique compression effect, superior bending performance, and sound insulation capabilities compared to traditional honeycomb sandwich structures, and it has garnered significant attention from researchers. Griese [27] et al. conducted a systematic, parametric study on the sound transmission and vibration characteristics of honeycomb core sandwich panels and re-entrant NPR sandwich panels using the structural acoustic finite element analysis method. It was observed that reducing the internal angle of the honeycomb sandwich while maintaining a constant mass constraint resulted in a decrease in its natural frequency and increased resonance in the 1–1000 Hz range, as well as an increase in the sound pressure transmission loss between the resonance frequencies. Zhang [28] et al. discovered that the re-entrant NPR sandwich structure exhibited superior vibration isolation performance compared to the honeycomb structure. At the same time, they also found that reducing its relative density and increasing its Poisson’s ratio can enhance the vibration isolation capability of the structure. Li [29] et al. expanded the novel sandwich structure by incorporating re-entrant hexagons, regular hexagons, and hybrid honeycomb sandwich structures. They found that, by adjusting the ratio and position of the three sandwich structures in the sandwich panel, the bending performance, fundamental frequency, and sound insulation performance of the sandwich structure can be improved, allowing it to obtain better sound transmission loss performance than the traditional sandwich honeycomb structure. Li and Yang [30] discovered that the uniform and gradient auxetic double arrowhead honeycomb structure exhibited superior vibration attenuation and sound attenuation behavior compared to traditional honeycomb structures while also possessing higher bending stiffness. It was found that, by adjusting the relevant parameters of the sandwich structure, the vibration damping performance and noise control ability at specific frequencies were enhanced. Sheykhi [31] et al. discovered that the star-shaped NPR sandwich structure has better static bending compliance and vibration and sound attenuation performance in the overall structure than the traditional honeycomb structure. Based on the analysis of the influence of the parameters in the star-shaped NPR sandwich structure on its sound insulation performance, they optimized the sound transmission loss in a specific frequency range and improved the sound insulation performance of this frequency band by five times. Based on the hyperbolic tangent shear deformation theory, Li [32] et al. investigated the free vibration and sound insulation performance of two types of honeycomb sandwich panels with functional gradients of negative Poisson’s ratio. The results show that the honeycomb sandwich structure with negative Poisson’s ratio exhibits better sound insulation performance in the low frequency range.

Based on the aforementioned exposition, numerous scholars have extensively investigated the acoustic insulation capabilities of sandwich structures exhibiting negative Poisson’s ratios, thus affirming the substantial potential of such structures for applications in sound insulation. However, these investigations primarily focus on the sound insulation performance of sandwich structures under theoretical circumstances, exhibiting a dearth of experimental cross-validation and overlooking the variations in sound insulation efficacy observed under real-world conditions.

Based on the aforementioned issues, this study designs a sandwich metamaterial sound transmission loss structure based on the classic re-entrant negative Poisson’s ratio (re-entrant NPR sandwich structure) configuration. The physical prototypes of the proposed structure are fabricated using 3D additive manufacturing technology. The finite element modeling software COMSOL Multiphysics was employed to computationally simulate the sound insulation characteristics of the sandwich structure, while the acoustic impedance tube method was utilized to perform experimental assessments on physical specimens. The experimental results served to substantiate the accuracy and efficacy of the simulation outcomes, thereby validating the high fidelity of the numerical simulations. Furthermore, a comprehensive parametric analysis of the sandwich structure was performed via high-fidelity numerical simulations. We explored the influence of geometric parameters such as the panel thickness, core height, structural wall length, and Young’s modulus of materials on the sound transmission loss performance of the sandwich structure.

## 2. Materials and Methods

### 2.1. Geometric Design of the Re-Entrant NPR Sandwich Structure

**Figure 1:**
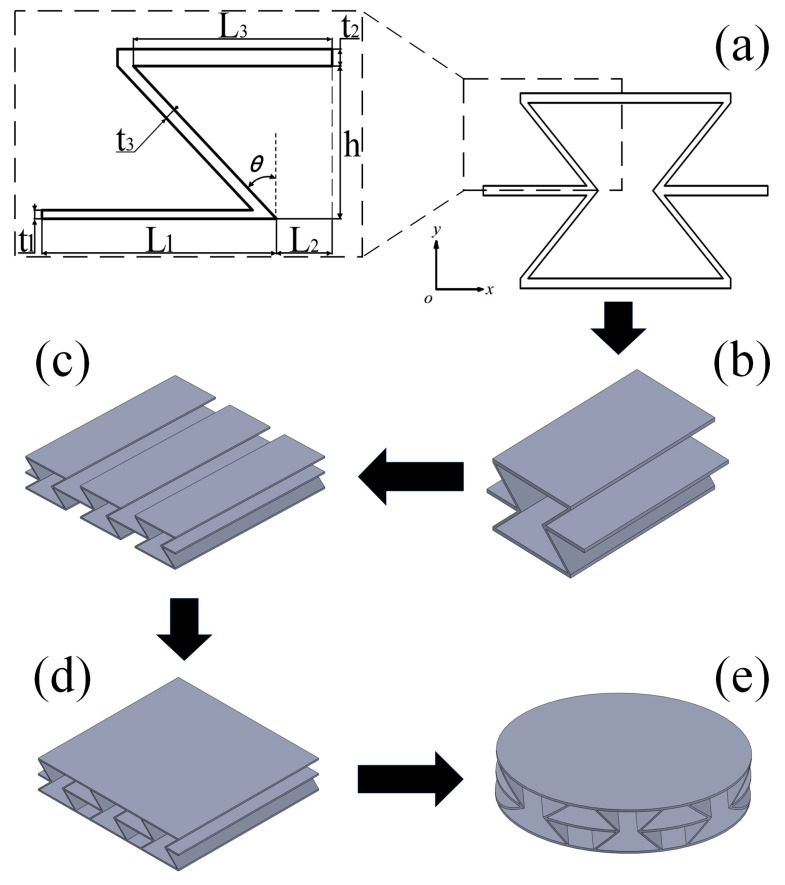
Geometric design of the sandwich structure: (**a**) Cross-sectional size diagram of the sandwich structure unit cell; (**b**) Sandwich structure unit cell; (**c**) Basic configuration of the sandwich structure; (**d**) Complete configuration of the sandwich structure; (**e**) The sandwich structure is cut out to test the specimen model used in the experiment.

The geometric configuration of the re-entrant NPR sandwich structure configuration discussed in this paper is shown in Figure 1a. The unit cell of the re-entrant NPR sandwich structure is composed of ribs; the length of the ribs is *L*_1_, the thickness of the ribs is *t*_1_, the distance between the ribs and the center of the unit cell is *L*_2_, the thickness of the upper and lower walls is *t*_2_, the length of the upper and lower walls is *L*_3_, the thickness of the side walls is *t*_3_, and the distance from the center of the cell to the upper and lower sides, and the distance from the wall, is *h*. For the re-entrant NPR sandwich structure, where each unit cell is connected by a rib plate, the dimensions of each unit cell are the same. Figure 1b is a sandwich structure unit cell. Figure 1d shows the combination of the upper and lower walls of multiple unit cells with the upper and lower panels to form a complete sandwich structure. Figure 1e is a sandwich structure with a radius of 50 mm, cut out to match the experimental conditions.

### 2.2. Mechanical Properties of the Re-Entrant NPR Sandwich Structure

Based on the theory of linear elasticity, Gibson [7] and others deduced the equivalent Poisson’s ratio analytical formula of the hexagonal honeycomb unit cell. This theory ignores the small variable of *t*/*L* (*t* is the wall thickness of the sandwich structure, and *L* is the length of the side wall, that is, the length of the wall where *t*_3_ is located), and does not consider axial deformation and shear deformation, but only pays attention to the bending deformation, which plays a major role.

(1) $$v_{yx}^{*}=-\frac{\varepsilon_{x}}{\varepsilon_{y}}=\frac{1}{v_{xy}^{*}}=\frac{\cos^{2}\theta }{(2L_{3}/L+\sin\theta )\sin\theta }$$

where $\varepsilon_{x}$ is the deformation of the sandwich structure in the *x* direction when the sandwich structure is compressed, $\varepsilon_{y}$ is the deformation of the sandwich structure in the *y* direction when the sandwich structure is compressed, $v_{xy}^{*}$ represents the Poisson’s ratio of the sandwich structure, *θ* is the angle between the side wall and the vertical line, and the clockwise direction is defined as positive. 

When *θ* < 0°, the unit cell has negative Poisson’s ratio characteristics; *L*, *θ*, and the structure. The geometric relationship of the parameters is:

(2) $$L=\sqrt{h^{2}+{\left(L_{1}+L_{2}\right)}^{2}}$$

(3) $$\theta =\arctan\frac{L_{3}-L_{2}}{h}$$

The formulas for calculating the equivalent Young’s modulus and equivalent shear modulus of the sandwich structure are:

(4) $$E_{x}^{*}v_{yx}^{*}=E_{y}^{*}v_{xy}^{*}=E_{x}(\frac{t}{L})\frac{1}{\sin\theta \cos\theta }$$

(5) $$G_{xy}^{*}=E_{x}{\left(\frac{t}{L}\right)}^{3}\frac{\left(h/L+\sin\theta \right)}{{\left(h/L\right)}^{2}(1+2h/L)\cos\theta }$$

where *E_x_* is the Young’s modulus of the structure material of the unit cell, and the wall thickness t of the re-entrant NPR sandwich structure is:

(6) $$t=(2t_{1}+t_{2}+2t_{3})/4$$

The relative density is an important parameter for controlling the stiffness, strength, and toughness of the sandwich structure. The relative density of the re-entrant NPR sandwich structure is:

(7) $$\rho =\frac{A_{s}}{A_{a}}=\frac{(L_{1}+L_{2})\times (h+t_{2})-(L_{1}-L_{4})\times L_{2}+(2L_{1}+2L_{2}-2L_{4})\times h/2}{\left(L_{1}+L_{2})\times (h+t_{2}\right)}$$

where *A_s_* represents the area occupied by the wall plate of the unit cell; *A_ɑ_* is the effective total area of the unit cell, and *L*_4_ is the length of the connecting surface between the side wall and the upper and lower walls:

(8) $$L_{4}=t_{3}/\cos\theta$$

### 2.3. Manufacture of Re-Entrant NPR Sandwich Structure Specimens

**Figure 2:**
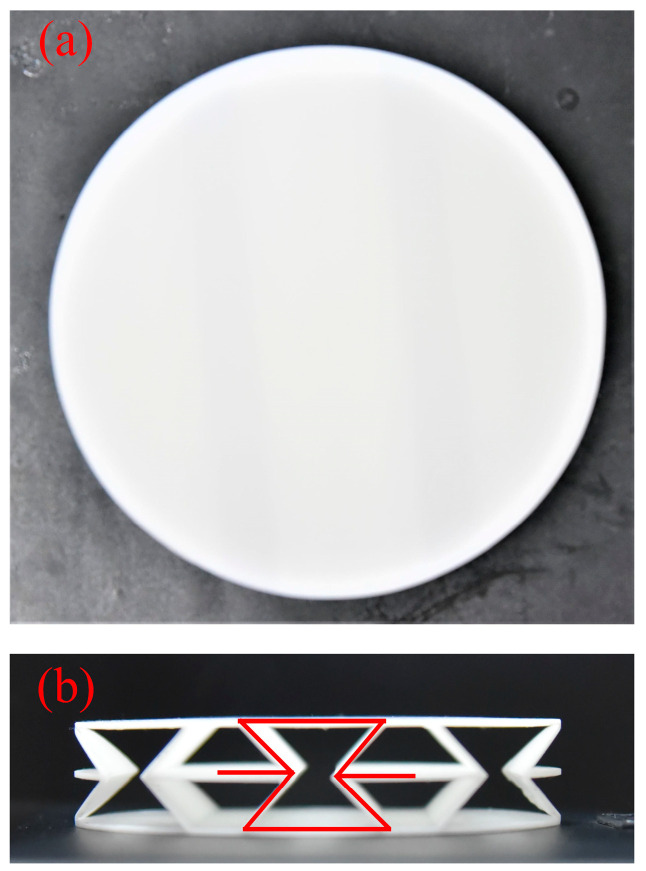
Sandwich structure specimen used for impedance tube experiment: (**a**) Top view of experimental specimen; (**b**) Front view of experimental specimen.

Figure 1 shows the construction flow chart of the sandwich structure. Considering the factors of the experimental equipment, the manufacturing size of the specimens is set as a cylinder with a radius of 50 mm. The corresponding experimental specimens were manufactured through 3D additive manufacturing technology (the machine model is China Taiwan ZRapid Tech Company Zhongrui SL660), and the material used in this additive manufacturing is photosensitive resin DSM8000. Table 1 shows the geometric parameters of the printed sandwich structure. The three-dimensional model diagram and the physical diagram are shown in Figure 1e and Figure 2.

### 2.4. Tensile Testing of Materials

**Figure 3:**
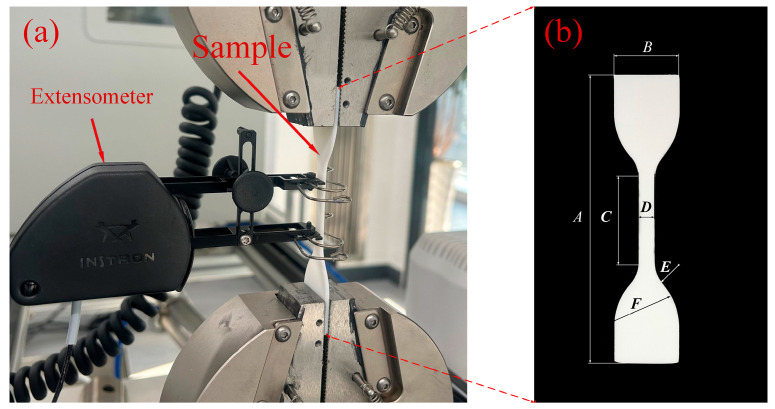
(**a**) Material elastic modulus test. (**b**) Structural diagram of the tensile test piece.

**Figure 4:**
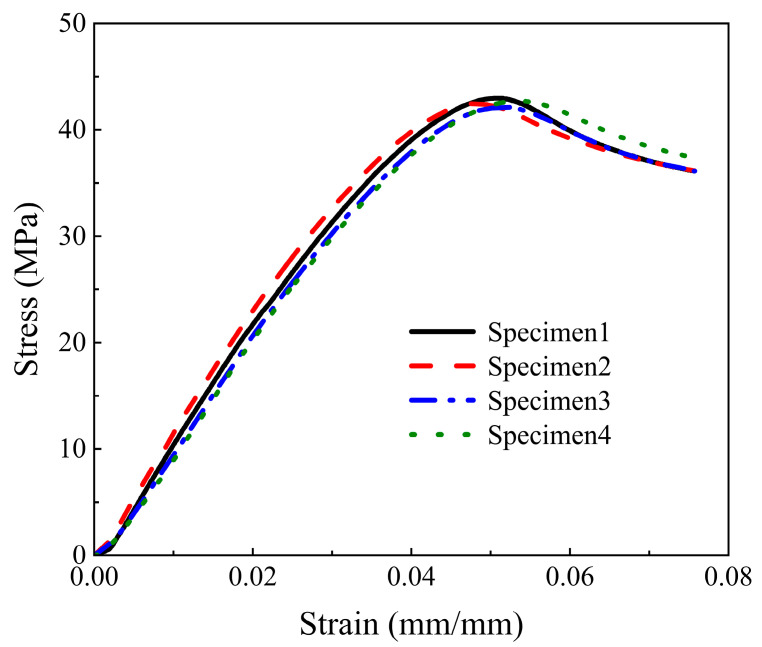
Tensile test stress–strain curve.

In order to obtain material performance data, a tensile test was carried out on the photosensitive resin material constituting the sandwich structure. In this tensile test, a universal mechanical testing machine (Instron Corporation, Boston, MA, USA model: INSTRON 3343) and an electronic extensometer (Instron Corporation, Boston, MA, USA model: Instron STATIC) were used. The tensile speed of the testing machine was controlled at 2 mm/min, and the test standard was the ISO37-2017 standard. Table 2 shows the geometric parameters of the specimen structure of this tensile test, the experimental shape is shown on the right side of Figure 3b. The geometric parameters of the tensile specimen are: overall length *A*, width of ends *B*, length of narrow portion *C*, width of narrow portion *D*, transition radius outside *E*, transition radius inside *F* and tensile test piece thickness *t* *. In order to reduce experimental errors, the specimens used in this tensile test and the sandwich structure specimens were fabricated together by the same machine. The diagram of the specimens and the experimental process are shown in Figure 3. Figure 4 is the stress–strain curve corresponding to the Young’s modulus of each specimen measured in this experiment. Table 3 shows the mass and Young’s modulus of each of the test pieces. The average Young’s modulus *Es* of the sandwich structure material measured in this test is 1040.65 MPa, Poisson’s ratio *v* is measured as 0.2, and the material density *ρ_m_* is calculated as 1.157 g/cm^3^. We will use these data in subsequent finite element simulations.

### 2.5. Finite Element Modeling (FEM)

#### 2.5.1. Model Analysis

**Figure 5:**
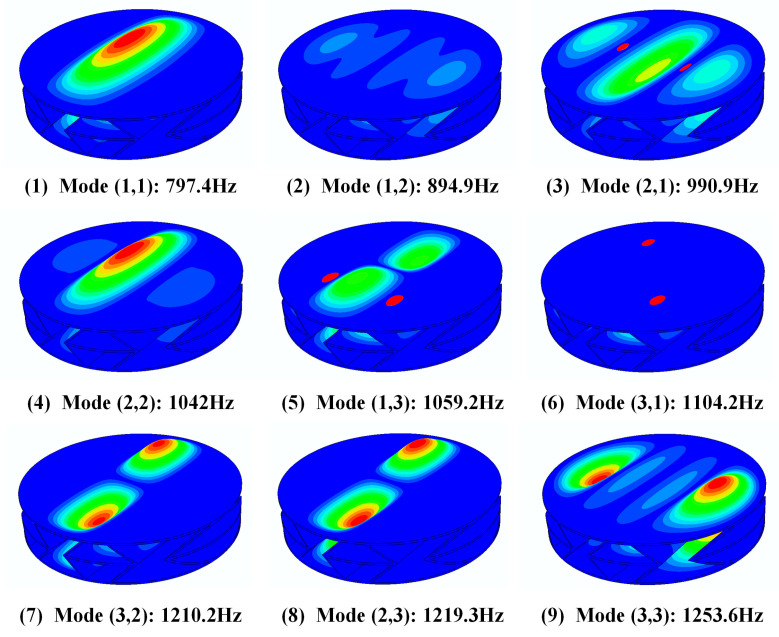
The first 9 natural frequencies of the sandwich structure.

The first nine natural frequencies of the sandwich structure were obtained through numerical simulation using the finite element analysis software ABAQUS 6.13. Resonance occurs when the frequency of an incident sound wave matches the natural frequency of a material. In sound insulation applications, this may cause the energy of sound waves to travel through the material more easily, thereby reducing the sound insulation effect. In addition, the resonance may also induce vibrations within the material itself, generating noise which further impacts the sound insulation performance. Table 4 shows the resonant frequencies corresponding to the first 9 modes of the sandwich structure, and Figure 5 shows the cloud diagram of the force corresponding to the 9 modes. When the direction and frequency of the incident sound wave corresponds to the vibration mode of the mode, this will cause the resonance of the sandwich structure, thereby generating a sound insulation valley. We will compare and analyze the natural frequency of the sandwich structure obtained through simulation with the subsequent acoustic simulation results.

#### 2.5.2. Acoustic Simulation

**Figure 6:**
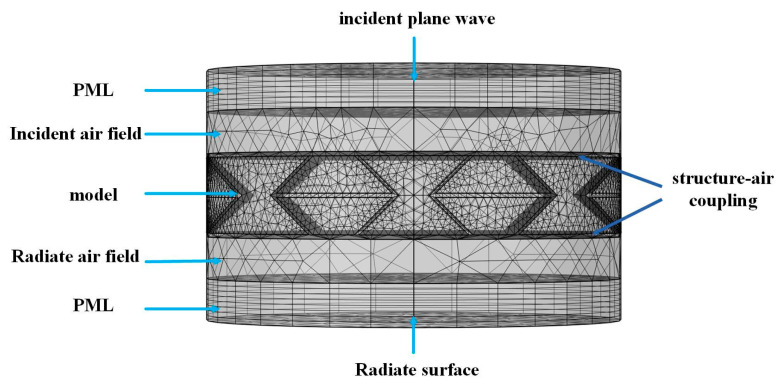
The FE model with free boundary conditions.

As shown in Figure 6, the corresponding model is established in the finite element software COMOL Multiphysics 5.4, and uses the two modules “Pressure Acoustics” and “Solid Mechanics”. Import the established 3D model of the sandwich structure into the software of COMOL Multiphysics, establish the incident air field and radiate air field, Perfectly Matched Layers (PML), set the corresponding material parameters and configuration parameters, and divide the mesh. The sound wave is incident on the radiation surface in the form of plane waves from the front end of the incident air field, passes through the radiate air field, and radiates completely in the form of Perfectly Matched Layers. Simulate the conditions of the sandwich structures in acoustic impedance tubes. The edge of the plate is set as a free boundary, the sound pressure of the incident sound wave is set to 1 Pa, the sound velocity is 343 m/s, the air density is 1.29 kg/m^3^, the sandwich structure is vertically incident in the form of a plane wave, and the frequency band of the incident sound wave is 250–1600 Hz.

Acoustic simulation using numerical methods has special requirements for the mesh size. For linear finite element and boundary element models, the largest element typically needs to be smaller than 1/6 the wavelength of the highest computational frequency. When dividing the sandwich element mesh, a larger element size will affect the calculation accuracy of the finite element, and an element size that is too small will increase the calculation amount and calculation time. In order to obtain accurate calculations, it is necessary to ensure that more than 6 elements are included in one wavelength unit. Therefore, considering the efficiency and accuracy of the finite element calculation, the mesh size of the sandwich structure is about 1 mm, the mesh size of the front and rear air layers is 1 mm, and the Perfectly Matched Layers is set to an array of 8-layer grids.

### 2.6. Experimental Device

In this paper, the acoustic impedance tube test method is used to study the STL performance of the sandwich structure. Acoustic impedance tube measurement is an experimental method commonly used to characterize the acoustic properties of materials or structures, and is usually used to measure the propagation and reflection of sound waves at material interfaces or in pipes. To a certain extent, the acoustic impedance tube experiment will be limited by the frequency caused by the tube size effect, and, at the same time, there are certain restrictions on the size and structure of the test specimen. During the experiment, one end of the acoustic impedance tube is used to emit sound waves perpendicular to the surface of the specimen in order to measure the sound transmission loss of the sandwich structure under the incidence of plane waves. Although the acoustic impedance tube measurement has certain limitations, its measured results are still accepted, and it has become one of the commonly used tools for measuring the sound insulation performance of structures [33,34,35,36].

**Figure 7:**
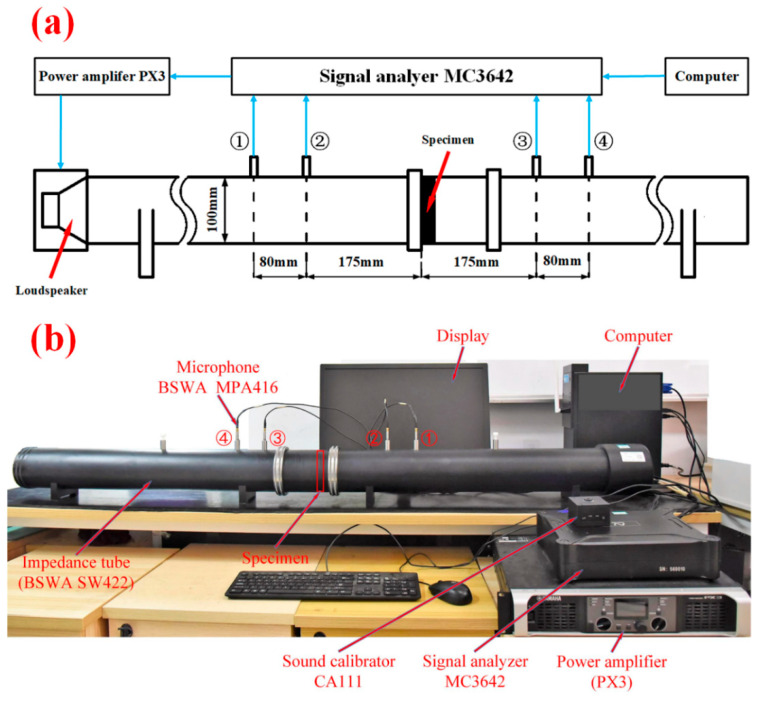
(**a**) Schematic diagram of the experimental system; (**b**) Physical diagram of the acoustic impedance tube experimental equipment.

The schematic diagrams of the acoustic impedance tube test method and the actual test equipment are shown in Figure 7a and Figure 7b, respectively. The computer software can control the signal analyzer (China Beijing BSWA TECH Company machine Model: MC 3642) to generate parallel sound waves, which are amplified by the power amplifier (China Beijing BSWA TECH Company machine Model: PX3) within the frequency power of 200–1600 Hz. Sound is output from the speaker of the acoustic impedance tube (China Beijing BSWA TECH Company machine Model: SW422) into the tube. Then, the signal analyzer uses 4 microphones (China Beijing BSWA TECH Company machine Model: MPA416) to collect the sound signal, and the microphones transmit it to the computer software for processing in order to obtain the STL value of the sandwich structure.

It is worth mentioning that the four microphones, ①, ②, ③, and ④, need to be connected to the corresponding four channels on the signal analyzer (as shown in Figure 7). In addition, the distances from ① to ② and from ③ to ④ must be consistent; all must be 80 mm. The distance from microphones ② and ③ to the left side of the specimen is 175 mm.

Before the experiment process starts, the sound pressure needs to be calibrated with the sound calibrator (Model:CA111), and the internal transfer function of the software is automatically modified to achieve calibration. During the experiment, place the specimen in the corresponding impedance tube. Each experiment is divided into two parts. One part is the measurement of the closed back cover, and the other part is the measurement of the open back cover. Then, the sound insulation data of the current experiment are automatically calculated by the software. The average value of multiple (>5) repeated measurements was taken as the final sound insulation data of this group of experiments. Multiple sets of experimental measurements were carried out on the same batch of test pieces, and, finally, the sound insulation data of the sandwich structure was obtained.

### 2.7. Sound Transmission Loss Performance of Re-Entrant NPR Sandwich Structure

**Figure 8:**
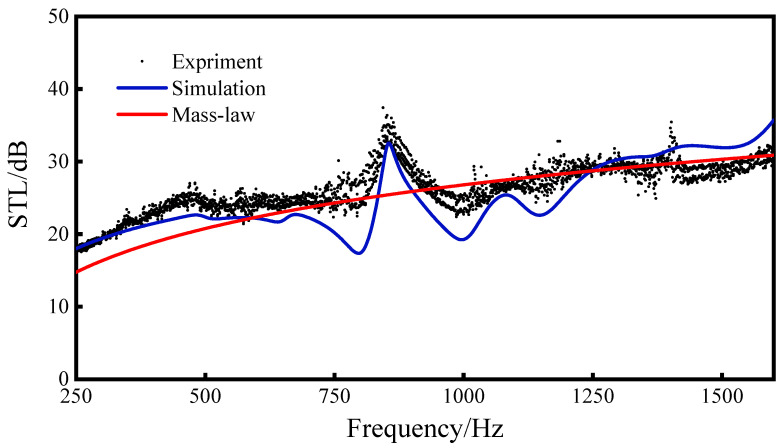
Comparison between experiment and simulation of re-entrant NPR sandwich structure sound transmission loss.

Through a comprehensive analysis of the impedance tube experiment and the numerical simulation results of the sandwich metamaterial sound insulation structure based on the re-entrant NPR configuration (as depicted in Figure 8), it is evident that the simulation and experimental outcomes exhibit a satisfactory level of agreement. While slight differences exist between the simulation and experimental results, the overall trends align closely, and the discrepancies between the two remain within an acceptable range of error. There may be the following reasons for the difference: (1) The boundary conditions of the specimen installation are not perfect. This experiment is carried out in the form of imitating the free boundary, and a small gap is left between the sandwich structure and the tube wall. Vaseline is applied between the impedance tube and the sandwich structure in order to prevent sound leakage, but this will cause a certain amount of friction and stress in the sandwich structure, making it difficult to achieve the effect of a truly free boundary. (2) There are some errors in the specimen’s manufacturing.

Based on the results of the simulation experiment, we can conclude that the simulation and the experiment have a good consistency, and the experiment verifies that the finite element simulation performed by the COMOL Multiphysics software has high fidelity.

Through the analysis of the experimental and simulation results, it can be seen that the sound insulation valleys of the sandwich structure correspond to the natural frequencies (1,1), (2,1), and (3,1) of the structure at 798 Hz, 996 Hz, and 1146 Hz, respectively. Through the analysis of the experimental and simulation results, it can be seen that the sound insulation valleys of the sandwich structure are close to the natural frequencies (1,1), (2,1), and (3,1) of the structure at 798 Hz, 996 Hz, and 1146 Hz, respectively. This is because the main vibration direction of the above-mentioned third-order mode is consistent with the incident direction of the sound wave. When the frequency of the sound wave is close to the natural frequency of the structure, the corresponding mode will be excited, causing the structure to resonate and generate a sound insulation valley.

According to the sound power calculation formula:

(9) $$STL=10\mathrm{lg}(\frac{w_{i}}{w_{t}})$$

In the formula, *w_i_* and *w_t_* represent the sound power of the incident sound wave and the transmitted sound wave, respectively.

It can be seen from the above formula that the sound transmission loss of 20 dB means that, after the sound wave passes through the sandwich structure, the energy is reduced by 99%, which proves that the re-entrant NPR sandwich structure has good sound insulation performance. The sound transmission loss of the sandwich structure at 250–1600 Hz can basically reach 20 dB, and the highest sound transmission loss can reach 35 dB. In the experiment, the average sound insulation of 250–1600 Hz was 26.29 dB, and, in the simulation, the average sound insulation of the sandwich structure in the range of 250–1600 Hz reached 24.87 dB. Both reached the level of the average sound insulation (25.17 dB) of the mass law, indicating that the re-entrant NPR sandwich structure reached the average sound insulation of the mass law (25.17 dB) level.

Due to the limitation of experimental conditions, this experiment can only verify the sound transmission loss effect in the frequency range of 250–1600 Hz, but the consistency between simulation and experiment in the frequency range of 250–1600 Hz is better. At the same time, some relevant personnel have verified that the simulation and the acoustic impedance tube experiment have good fidelity [33,34,37]. Therefore, it is determined that the simulation results can represent the real sound insulation effect. 

**Figure 9:**
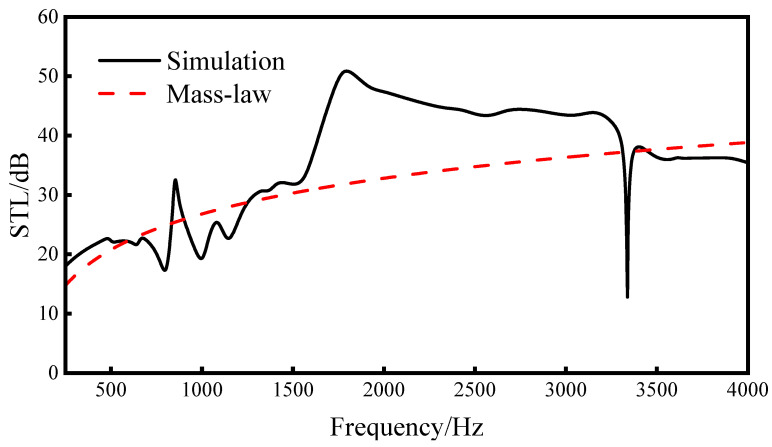
Comparison of the re-entrant NPR sandwich simulation sound transmission loss curve and mass theorem curve.

Given that the primary noise frequency bands of high-speed trains tend to concentrate within the range of 250–4000 Hz [1,3], the frequency detection range for this research is set accordingly. Figure 9 illustrates that the average sound transmission loss within the frequency range of 250–4000 Hz achieved a substantial value of 36.0 dB. Notably, this result indicates a pronounced suppression effect in the noise frequency band (1000–4000 Hz), which corresponds to the range that the human ear is particularly sensitive to. Compared with the average sound insulation of the quality theorem in the same frequency range of 31.7 dB, it is 4.3 dB higher, and, especially in the frequency range of 1655–2405 Hz, the sound transmission loss of this sandwich structure is more than 10 dB higher than the sound transmission loss of the mass theorem at the same frequency. It shows that the re-entrant NPR sandwich structure is better than the solid structure with the same volume within the research range. Therefore, the re-entrant NPR sandwich structure realizes the functions of being light weight and having high sound transmission loss performance.

## 3. Parameter Analysis

In the previous section, we confirmed that the acoustic simulation of the sandwich structure using COMOL Multiphysics 5.4 has high fidelity, and the simulated structure can represent the experimental results within a certain range. In this section, we will use the verified FEM to conduct parametric research on the specimens and explore the influence of different structural parameters on the sound insulation effect of the specimens. The parameters of this study include rib thickness *t*_1_, rib length *L*_1_, distance from rib to cell center *L*_2_, upper and lower wall thickness *t*_2_, upper and lower wall length *L*_3_, Side Wall Thickness *t*_3_, distance from cell center to upper and lower walls *h*, and material Young’s modulus *E_x_*. The structural parameters of the reference group for parameter analysis are: *L*_1_ = 14.75 mm, *L*_2_ = 3.5 mm, *L*_3_ = 12.5 mm, *t*_1_ = 0.5 mm, *t*_2_ = 1 mm, *t*_3_ = 0.7 mm, and *h* = 9 mm. During parameter analysis, only one group of parameters is changed, and the remaining parameters are kept unchanged. In addition, the increase and decrease in the analyzed parameters are kept at 20%.

### 3.1. Thickness of Ribs t_1_

Keeping *L*_1_, *L*_2_, *L*_3_, *L*_4_, *t*_2_, *t*_3_, *h*, and *E_x_* unchanged, by changing the thickness *t*_1_ of the rib plate, analyze the sound transmission loss of the sandwich structure when *t*_1_ = 0.4 mm, *t*_1_ = 0.5 mm, and *t*_1_ = 0.6 mm.

**Figure 10:**
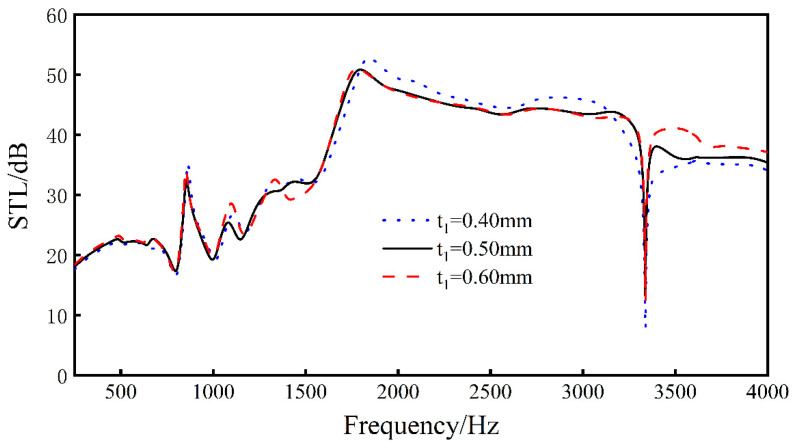
Effect of different rib thicknesses on the sound transmission loss performance of sandwich structures.

As can be seen from Figure 10, changing the thickness of the ribs of the sandwich structure within a certain range will hardly change the sound transmission loss curve of the structure, indicating that the change of the thickness of the ribs will not have a great impact on the sound insulation of the sandwich structure.

### 3.2. Ribs Length L_1_

Change the thickness *L*_1_ of the ribs, and analyze the sound transmission loss of the sandwich structure when *L*_1_ = 11.8 mm, *L*_1_ = 14.75 mm, and *L*_1_ = 17.7 mm.

**Figure 11:**
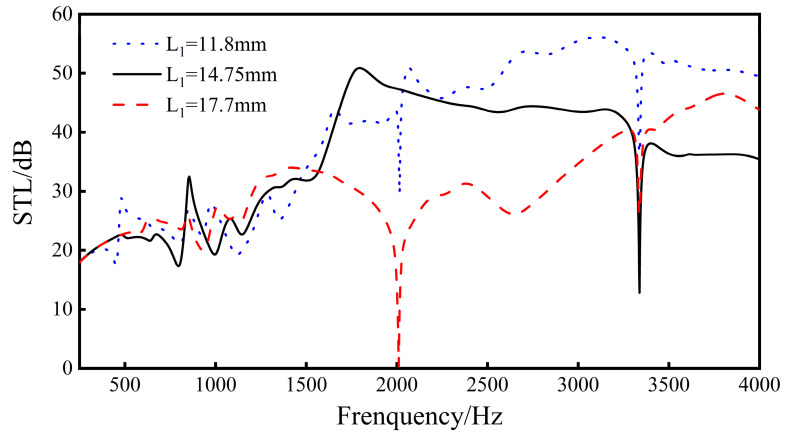
Effect of different rib lengths on the sound transmission loss performance of sandwich structures.

**Figure 12:**
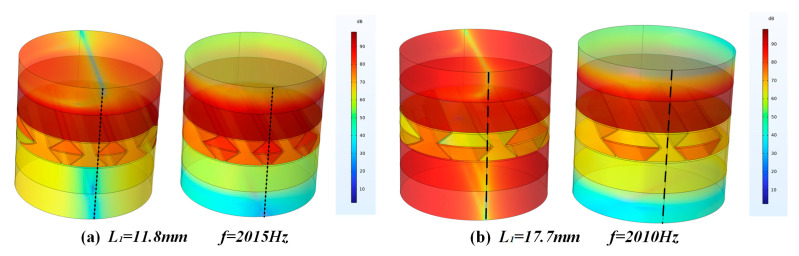
(**a**) The sound pressure level diagram of the frequency where the troughs of the curves of *L*_1_ = 11.8 mm (**b**) The sound pressure level diagram of the frequency where the troughs of the curves of *L*_1_ = 17.7 mm.

Increasing the length *L*_1_ of the ribs will have a greater impact on the sound transmission loss effect of the sandwich structure. As shown in Figure 11, when the length of the ribs increases, the distance between the unit cells in the sandwich structure will increase, and the space above and below the ribs of the unit cells will increase. At the same time, the sound insulation of the sandwich structure in the middle and high frequency range (1500–4000 Hz) decreases with the increase in the rib length, and the overall sound insulation gradually decreases. For the frequency band below 1500 Hz, the sound insulation trend is relatively flat with the increase in the rib length, and the overall sound insulation does not change much. In addition, when the rib length is 11.8 mm and 17.7 mm, both of them produce a new sound insulation valley at 2010 Hz. It can be seen from Figure 12 that, under the same condition of *L*_1_, when the side ribs of the re-entrant NPR sandwich structure are located at the center of the circle, as shown in Figure 12a, a corresponding sound insulation trough will be generated. When the ribs are not in the center of the circle, as shown in Figure 12b, the acoustic valleys disappear. It can be seen from the figure that the reason for the sound insulation valley is that the circular structure used in the finite element simulation and the re-entrant NPR sandwich structure produces phase interference at the corresponding frequency, resulting in the sound insulation valley.

The results show that the sound insulation of the negative Poisson’s ratio sandwich structure is greatly affected by the length of the ribs. Within a certain range, with the increase in the length of the ribs, the overall sound insulation gradually attenuates. Therefore, in actual use, one should use caution when selecting the rib length for the sandwich structure.

### 3.3. Upper and Lower Wall Thickness t_2_

**Figure 13:**
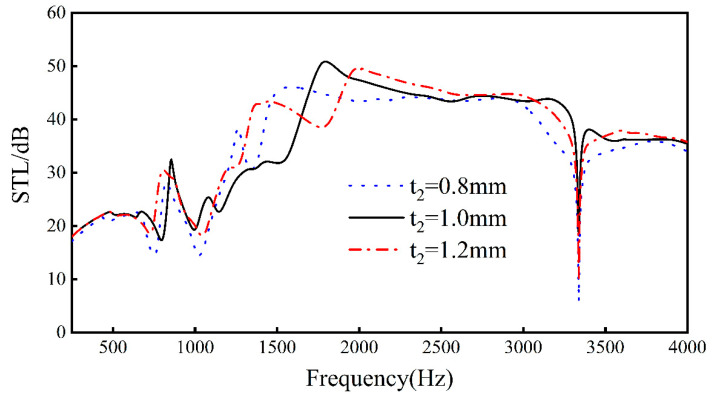
Effect of different rib thicknesses on the sound transmission loss performance of sandwich structures.

Keeping *L*_1_, *L*_2_, *L*_3_, *L*_4_, *t*_1_, *t*_3_, *h*, and *E_x_* unchanged, by changing the thickness *t*_2_ of the rib, analyze the change of sound transmission loss when *t*_2_ = 0.8 mm, *t*_2_ = 1.0 mm, and *t*_2_ = 1.2 mm. The results can be seen in the Figure 13 below.

Increasing the thickness of the upper and lower walls only slightly increases the overall sound insulation of the structure, but has a greater impact on the frequency of the peaks and valleys of the sandwich structure and the sound insulation. This is because increasing the thickness of the upper and lower walls will increase the stiffness and overall quality of the sandwich structure, resulting in a change in the natural frequency of the sandwich structure. In the frequency range of 250–3338 Hz, with the increase in the upper and lower ratio thickness, the overall sound insulation will first increase and then decrease, but, when the frequency exceeds 3338 Hz, the sound insulation will be slightly attenuated with the increase in thickness. In addition, we also found that the thicker the upper and lower walls are, the slower the valleys-to-peaks trend.

For the sound insulation valleys, when the thickness increases from 0.8 mm to 1.0 mm, the frequency at the first trough increases by 50 Hz, the frequency at the second trough decreases by about 20 Hz, and the sound insulation of the sound insulation trough increases by about 5 dB. When the thickness increases from 1.0 to 1.4 mm, for every 0.2 mm increase, the frequency at the first sound insulation valley decreases by about 60 Hz, and the frequency at the second trough increases by about 10 Hz. For the sound insulation peak, when the wall thickness of the upper and lower walls increases from 0.8 mm to 1.2 mm, each increase is 0.2 mm. The frequency of the re-entrant NPR sandwich structure at the sound insulation peak in the 1400–2700 Hz frequency band increases by about 250 Hz. At the same time, as the thickness of the upper and lower walls increases, the maximum peak value increases first and then decreases, reaching a maximum value of 50.9 dB at *t*_2_ = 1.0 mm. When the thickness reaches 1.4 mm, the re-entrant NPR sandwich structure splits from one peak to two peaks in the peak frequency bands of 670–1050 Hz and 1750–2240 Hz.

Therefore, we can draw a conclusion: when the thickness of the upper and lower walls increases, in the 250–4000 Hz frequency band, the overall sound insulation will first increase and then decrease with the increase in thickness, and the sound insulation peak will move to high frequency, increasing from the trough to the peak, and then trend will gradually slow down. In engineering applications, when it is necessary to reduce noise at a specific frequency, the sound insulation peak can be moved to this frequency by adjusting the upper and lower wall thicknesses, or the maximum overall sound insulation of the sandwich structure can be obtained through optimization.

### 3.4. Distance L_2_ from the Rib Plate to the Center of the Cell

Change the thickness *L*_2_ of the ribs, and analyze the re-entrant NPR sound transmission loss of the sandwich structure when *L*_2_ = 2.8 mm, *L*_2_ = 3.5 mm, and *L*_2_ = 4.2 mm.

**Figure 14:**
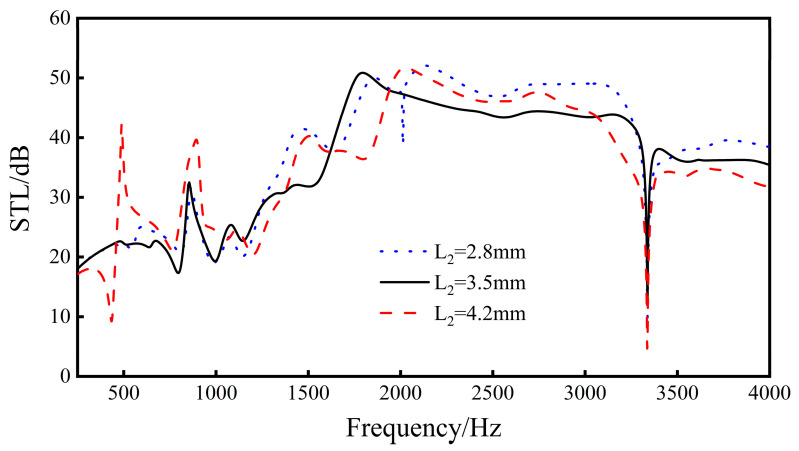
Effect of different rib distances on the sound transmission loss performance of sandwich structures.

Increasing the distance *L*_2_ between the rib plate and the center of the cell will increase the cavity volume inside the unit cell and increase the distance between the unit cells. As the distance between ribs increases, the sound insulation increases slightly. With the increase in the distance between ribs, the peak of sound insulation becomes higher, the valley of sound insulation gradually decreases, and the waveform fluctuation between the peak of sound insulation and the valley of sound insulation becomes larger and larger. It can be seen from Figure 14 that when *L*_2_ = 2.8 mm, a new sound insulation valley is generated at 2012 Hz, and the sound insulation is 38.8 dB. When *L*_2_ = 4.2 mm, new troughs and peaks are produced at 434 and 490 Hz.

The results show that, when the distance from the rib plate to the center of the cell increases from 2.8 mm to 4.2 mm, the overall sound insulation tends to increase slightly, but the fluctuation trend of the re-entrant NPR sound insulation curve increases with the increase in *L*_2_. In engineering applications, the influence of curve fluctuation should be considered so that the sound insulation trough of the sandwich structure avoids the main sound insulation frequency band.

### 3.5. Upper and Lower Wall Length L_3_

By changing the length *L*_3_ of the upper and lower walls, the re-entrant NPR sandwich structure-borne sound transmission loss when *L*_3_ = 10 mm, *L*_3_ = 12.5 mm, and *L*_3_ = 15 mm is obtained.

**Figure 15:**
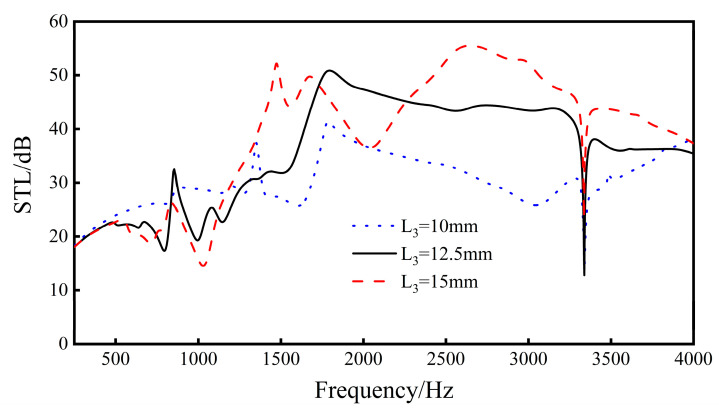
Effect of different lengths of the upper and lower walls on the sound transmission loss performance of the sandwich structure.

It can be seen from the Figure 15 that, when the length *L*_3_ of the upper and lower walls increases, the distance between the two sides of the sandwich structure unit cell increases, the volume of the internal cavity of the unit cell increases, the overall sound transmission loss of the structure increases, and the sound insulation wave peak moves to high frequency. The longer the length of the upper and lower walls, the greater the fluctuation of the sound insulation curve of the sandwich structure, especially in the middle and high-end frequency bands (1500–4000 Hz). 

The results show that, as the length *L*_3_ increases, the overall sound transmission loss of the sound insulation curve will increase, but the fluctuation of the curve will become larger and larger.

### 3.6. Side Wall Thickness t_3_

Keep *L*_1_, *L*_2_, *L*_3_, *L*_4_, *t*_1_, *t*_2_, *h*, and *E_x_* unchanged, and conduct parametric analysis on the side wall joint length *t*_3_. Explore the sound insulation effect when *t*_3_ = 0.56 mm, 0.7 mm, and 0.84 mm.

When the thickness of the side wall increases, the overall stiffness of the structure also increases, resulting in an increase in its natural frequency, and the peaks and valleys of the sound insulation wave shift to high frequencies.

**Figure 16:**
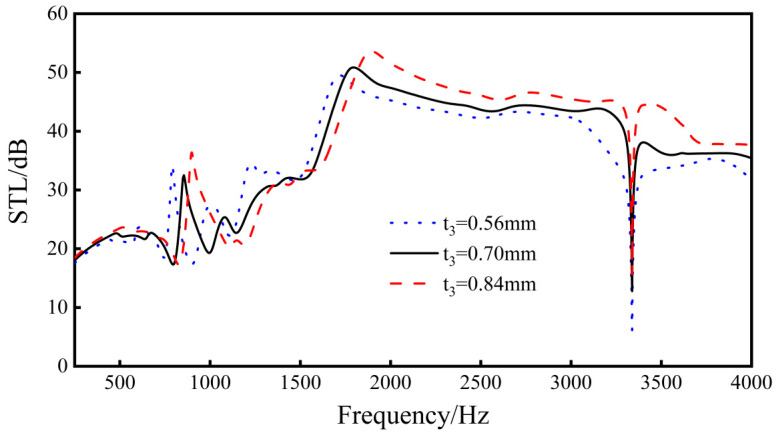
Effects of different sidewall connection lengths on the sound transmission loss performance of the sandwich structure.

It can be seen from Figure 16 that, when the thickness of the side wall increases, the trend of the overall sound insulation curve of the structure does not change much, but the overall sound insulation of the structure increases with the increase in the thickness of the side wall, which has a strong linear relationship. Among them, when *t*_3_ increases from 0.56 mm to 0.70 mm, the average sound insulation increases by 0.9 dB, the sound insulation at the sound insulation valley increases by 6.7 dB, and the peak frequency moves from 1716 to 1795 Hz. When *t*_3_ increases from 0.7 to 0.84 mm, the average sound insulation increases by 1.7 dB, the sound insulation of the sound insulation valley increases by 2.9 dB, and the peak frequency moves from 1795 to 1894 Hz.

From the results, as the length of *t*_3_ increases, the sound transmission loss performance of the sandwich structure increases. Therefore, the length of *t*_3_ should be as long as possible within a certain range.

### 3.7. The Distance h from the Center of the Cell to the Upper and Lower Walls

Keeping *L*_1_, *L*_2_, *L*_3_, *L*_4_, *t*_1_, *t*_2_, *t*_3_, and *E_x_* unchanged, the distance *h* between the center of the cell and the upper and lower walls is analyzed parametrically.

**Figure 17:**
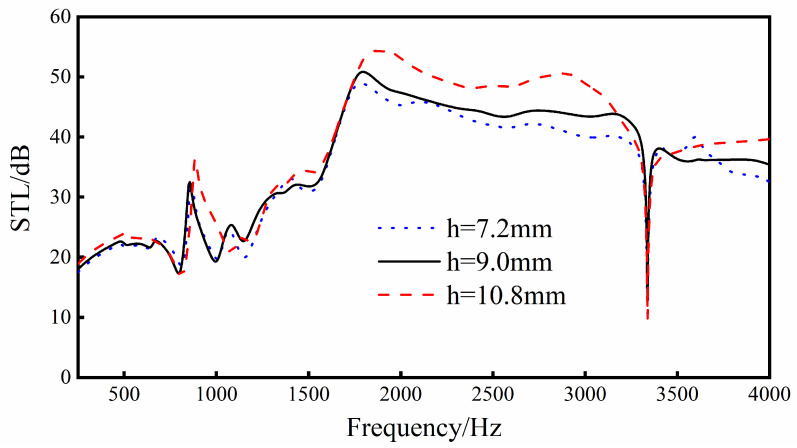
Effect of different distances from the center of the cell body to the upper and lower walls on the sound transmission loss performance of the sandwich structure.

It can be seen from Figure 17 that, when increasing the distance from the center of the cell to the upper and lower walls, the trend of the sound insulation curve of the sandwich structure will basically not change, but the overall sound transmission loss will increase. Especially in the middle and high frequency bands (1500–4000 Hz), the increase in sound transmission loss is more obvious, and there is a relatively large increase.

To sum up, in practical applications, increasing the distance from the center of the cell body to the upper and lower walls within a certain range can improve the overall sound insulation performance of the sandwich structure.

### 3.8. Young’s Modulus E_x_ of the Material

Keep *L*_1_, *L*_2_, *L*_3_, *L*_4_, *t*_1_, *t*_2_, *t*_3_, and *h* constant, and conduct parametric analysis on the Young’s modulus of the material. Discuss the trend of sound insulation when the Young’s modulus of the sandwich structure material is in the range of 832 to 1248 Mpa.

**Figure 18:**
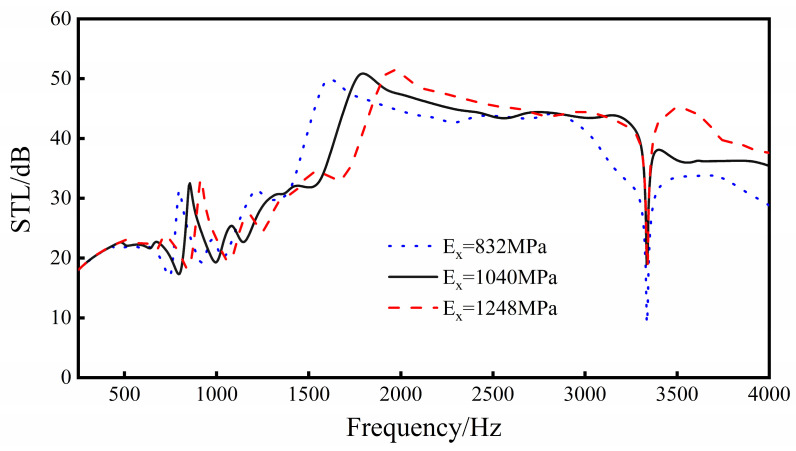
Effect of material Young’s modulus on sound transmission loss performance of the sandwich structure.

It can be seen from Figure 18 that, with the increase in Young’s modulus, the overall sound transmission loss of the sandwich structure increases. For every 20% increase in Young’s modulus, the average sound insulation of the sandwich structure in the 250–4000 Hz frequency range increases by about 1 dB. At the same time, for every 20% increase in Young’s modulus, the natural frequencies of the 1st, 3rd, and 6th orders (the first three sound insulation valleys) of the sandwich structure increase by 50, 70 and 110 Hz, respectively, and the frequencies of the two sound insulation peaks increase by 60 and 160 Hz, respectively. In addition, the increase in Young’s modulus has a significantly higher impact on the sound transmission loss performance of the sandwich structure in the frequency range of 1500 to 4000 Hz than in the frequency range of 250–1500 Hz.

Increasing the Young’s modulus of the material increases the structural rigidity, which increases the natural frequency of the sandwich structure, and the sound insulation peaks and valleys move to high frequencies. Therefore, when selecting materials, under the condition of satisfying the performance of structural mechanics, the Young’s modulus of the selected materials should be as large as possible.

## 4. Conclusions

In this paper, a sandwich metamaterial sound transmission loss structure is designed and manufactured based on the concave negative Poisson’s ratio configuration. The re-entrant NPR sandwich structure and the sound transmission loss (STL) performance of the proposed structure are investigated using a combination of numerical simulations and experimental methods.

1. There is a good consistency between the experiment and the simulation. The experiment and simulation show that the re-entrant NPR sandwich structure has a good sound insulation effect. In the explored frequency range (250–4000 Hz), the sound transmission loss can basically reach 20 dB, and the overall sound transmission loss level exceeds the sound transmission loss level of the mass theorem curve. The goals of good sound insulation effect and light weight have been achieved. The noise frequency band to which human ears are sensitive has an obvious suppression effect, especially in the frequency range of 1645–3290 Hz. The sound insulation exceeds 40 dB and has good application value in practical engineering applications.
2. Within this study, a comprehensive analysis is conducted on the geometric parameters of the re-entrant NPR sandwich structure, as well as the Young’s modulus of the material. The analysis reveals the following key findings: The sound transmission loss performance of the re-entrant NPR sandwich structure is predominantly influenced by three parameters: the length of the rib plate (*L*_1_), the distance from the rib plate to the center of the cell body (*L*_2_), and the length of the upper and lower walls (*L*_3_). Increasing the values of these three parameters leads to increased fluctuations in the corresponding sound insulation curve. The thickness of the ribs (*t*_1_), the thickness of the upper and lower walls (*t*_2_), the thickness of the side walls (*t*_3_), the distance from the center of the cell to the upper and lower walls (*h*), and the Young’s modulus of the material (*E_x_*) exhibit relatively minor influences on the sound transmission loss performance of the sandwich structure. Modifying the values of these parameters does not yield significant variations in the sound insulation performance of the sandwich structure. However, it can still result in incremental improvements or reductions within a certain range, thereby affecting the overall sound insulation capability of the structure.

Moving forward, our research endeavors will conduct further exploration from three aspects: theory, numerical simulation and experiment, and developing applications in engineering. 

## Figures and Tables

**Table 1:** Geometric parameters of the sandwich structure.

Sample	*L*_1_	*t*_1_	*L*_2_	*t*_2_	*L*_3_	*t*_3_	*h*
Value	14.75 mm	0.5 mm	3.5 mm	1.0 mm	12.5 mm	0.7 mm	9.0 mm

**Table 2:** Tensile specimen parameters.

Parameter	Overall Length *A*	Width of Ends *B*	Length of Narrow Portion *C*	Width of Narrow Portion *D*	Transition Radius Outside *E*	Transition Radius Inside *F*	Thickness *t* *
Value	115 mm	25 mm	33 mm	6.2 mm	14 mm	25 mm	2 mm

**Table 3:** Young’s modulus and mass of tensile specimens.

Parameter	Mass	Young’s Modulus
Specimen1	4.3 g	1055.81 MPa
Specimen2	4.2 g	1066.13 MPa
Specimen3	4.3 g	1026.14 MPa
Specimen4	4.2 g	1014.37 MPa
Average	4.26 g	1040.65 MPa

**Table 4:** The first 9 natural frequencies of the sandwich structure.

Number	Mode (m,n)	Numerical Solution (Hz)
1	(1,1)	797.4
2	(1,2)	894.9
3	(2,1)	990.9
4	(2,2)	1042
5	(1,3)	1059.2
6	(3,1)	1104.2
7	(3,2)	1210.2
8	(2,3)	1219.3
9	(3,3)	1253.6

## Data Availability

This article details the data and results covered by this study.

## Abbreviation

Parameter	Definition
*t*_1_	Rib thickness
*L*_1_	Rib length
*t*_2_	The thickness of the upper and lower walls
*L*_2_	The distance between the ribs and the center of the unit cell
*t*_3_	The thickness of the side walls
*L*_3_	The length of the upper and lower walls
*L_4_*	The length of the connecting surface between the side wall and the upper and lower walls
*h*	The distance from the center of the cell to the upper and lower sides and the distance from the wall
*t*	The wall thickness of the unit cell structure
*L*	The length of the side wall
*θ*	The angle between the side wall and the vertical line
$\varepsilon_{x}$	The deformation of the sandwich structure in the x direction when the sandwich structure is compressed
$\varepsilon_{y}$	The deformation of the sandwich structure in the y direction when the sandwich structure is compressed
$v_{xy}^{*}$	The Poisson’s ratio of the sandwich structure
*A_s_*	The area occupied by the wall plate of the unit cell
*A_ɑ_*	The effective total area of the unit cell
$E_{x}^{*},E_{y}^{*}$	The equivalent Young’s modulus of the sandwich structure in the *x* and *y* directions
*E_x_*	The Young’s modulus of the structure material of the unit cell
$G_{xy}^{*}$	The equivalent shear modulus of the sandwich structure
*ρ*	The relative density of the re-entrant NPR sandwich structure
*w_i_*	The sound power of incident sound waves
*w_t_*	The transmitted sound wave sound power
*STL*	The sound transmission loss
*A*	Overall length of tensile test piece
*B*	Width of ends of tensile test piece
*C*	Length of narrow portion of tensile test piece
*D*	Width of narrow portion of tensile test piece
*E*	Transition radius outside of tensile test piece
*F*	Transition radius inside of tensile test piece
*t**	Tensile test piece thickness of tensile test piece
